# Air Monitoring Stations Far Removed From Drilling Activities Do Not Represent Residential Exposures to Marcellus Shale Air Pollutants. Response to the Paper by Hess et al. on Proximity-Based Unconventional Natural Gas Exposure Metrics

**DOI:** 10.3390/ijerph17020504

**Published:** 2020-01-13

**Authors:** Jonathan J. Buonocore, Joan A. Casey, Rachel Croy, John D. Spengler, Lisa McKenzie

**Affiliations:** 1 Center for Climate, Health, and the Global Environment, Harvard T.H. Chan School of Public Health, Boston, MA 02115, USA; 2 Environmental Health Sciences, Mailman School of Public Health, Columbia University, New York, NY 10027, USA; jac2250@cumc.columbia.edu; 3 Department of Environmental Health, Harvard T.H. Chan School of Public Health, Boston, MA 02115, USA; rcroy@hsph.harvard.edu (R.C.); SPENGLER@hsph.harvard.edu (J.D.S.); 4 Environmental and Occupational Health, Colorado School of Public Health, University of Colorado Denver, Denver, CO 80204, USA; Lisa.mckenzie@ucdenver.edu

## Introduction

In their study “Assessing Agreement in Exposure Classification between Proximity-Based Metrics and Air Monitoring Data in Epidemiology Studies of Unconventional Resource Development” Hess et al. perform a validation of the well-activity (WA) proximity models used in epidemiologic research studies of unconventional natural gas development (UNGD) [1]. The study lacks sufficient evidence to support the authors’ conclusion that there is significant exposure misclassification in the epidemiology that finds health effects around unconventional oil and gas (O&G) wells. Major flaws in the Hess et al. study’s validation method include inappropriate selection of air pollution data, missing information on other air pollution sources and meteorology, inappropriate choice of statistical methods, and misrepresentation of the purpose of proximity metrics used in epidemiological studies. These substantial shortcomings negate the study’s ability to answer the question: does a WA proximity metric correlate with actual levels of air pollutants in a region with UNGD? 

## Distance Between Air Monitoring Sites and Wells: A Mismatch of Geographical Scale

A number of issues with the Hess et al. study stem from the large distance between most of the air monitoring stations and natural gas wells in Pennsylvania. Air pollutant measures from locations within a few kilometers of natural gas drilling sites would be most essential to proximity WA model validation for estimating exposures to air pollutants [2]. However, the closest distance between the air monitor and natural gas wells in the Hess et al. study range from 0.3 km to 56 km, the median distances range from 100 to 250 km, and the maximum distances exceed 400 km (Hess et al. Figure 4 and Table 2). 

Hess et al. argue that the monitor locations are suitable surrogates for home residences, as would be the case in much of the O&G proximity epidemiology. Given that the 100–250 km median distances between wells and the monitors serving as surrogates, this assumption seems implausible. Additionally, most of the air monitoring sites used in the study do not represent the potential exposure to Marcellus Shale-associated air pollutants for the over one million Pennsylvanians living within 1.6 km of an active oil or gas (O&G) well [3]. 

The epidemiological studies that have used the proximity WA or similar models have all observed the greatest health implications in the densest areas of O&G well activity, generally at residences within 20 km of a well [2,4,5,6,7,8,9,10,11,12,13,14,15]. McMullin et al. (2018) recently evaluated air pollution measured from monitors at distances from O&G wells ranging from 0.1 km to 1.13 km to the closest O&G site and found a number of VOCs, notably benzene, ethylbenzene, and n-nonane [16]. In order to successfully assess the validity of the WA metrics used in the existing epidemiological studies, the study should be performed at a comparable geographical scale. 

The distances used by Hess et al. do not match expected transport distances for many of the pollutants monitored. Benzene, SO_2_, NO_x_, and CO have fairly short atmospheric lifetimes and can only travel short distances, especially since these sources are close to the ground. For example, SO_2_ from point sources was recently found to have an atmospheric lifetime of 4–12 h [17]. This means that for the lowest median distance to an SO_2_ monitoring station, 119 km, the wind speed needed to be approximately 10–30 km/h along that vector for the monitor to detect it. Since wind speeds at 30m altitude are generally under this range, this renders SO_2_ from many of these wells undetectable to many of the monitors. In short, most of the air monitoring sites used in this validation study are incapable of detecting Marcellus Shale well-related air pollutants because they are located too far away. Furthermore, recent research by Schade and Roest using a regulatory monitor in Texas detected air pollution coming from the Eagle Ford shale [18]. The Schade and Roest study was done using methods that accounted for meteorology and other sources in the region, demonstrating the utility of these methods. If similar monitoring and analysis were performed in Pennsylvania, it may produce similar results.

## Misrepresentation of the WA Metrics Used in Epidemiologic Studies

Prior literature has not claimed the WA metric only captures air pollution exposure. In fact, the metric was designed to capture complex, multifactorial exposures, including air, water, noise, and light pollution as well as community disruption, psychosocial stress, increased traffic, and other potential impacts related to O&G activity [19]. Most prior epidemiologic studies note this [2,4,5,6,7,8,9,10,11,12,13,14,15] and acknowledge the limitations of using a WA metric. Therefore, Hess et al. have misinterpreted the purpose of the WA metric. They have made an “apples to oranges” comparison—or in this case, they compared six air pollutants, with differing fate and transport properties, to a multifactorial exposures and stresses captured by the WA metrics that vary both spatially and temporally and act over short distances and time scales. 

## Lack of Incorporation of Meteorology

The Hess et al. study does not incorporate meteorology in its analyses, which helps explain the variance and fluctuations of UNGD contaminants that may be of significant public health concern [1]. It is fairly standard practice to incorporate at least upwind/downwind relationships between pollutant sources and monitors, and successful monitoring at this distance would likely require collection of fine-scale meteorological data, and ideally back-trajectory analysis. Other potentially important metrics include cloud cover, wind speed, wind direction, and time of day. These meteorological metrics affect intensity, frequency, and duration of air pollutants [20]. For example, in Brown et al.’s 2015 study, a box model for air pollution dispersion in an area with O&G activity showed that air pollution levels increased at night when there is less air mixing [20]. Direct measurements in areas of intensive O&G activity indicate that nighttime ambient benzene concentrations are double daytime concentrations [21].

## Not Incorporating Other Sources of Monitored Air Pollution

The Hess et al. study also does not incorporate other air pollution sources that could be detected by these monitors. The pollutants which these air pollution monitors measure come from a number of sources common in the area, including vehicular traffic, combustion of coal and gas for electricity generation, and home heating, not to mention natural gas processing and existing conventional O&G wells. There is no mention of apportioning total air pollution to sources. If these other air pollution sources are not accounted and adjusted for, the study design used by Hess et al. should be unable to detect the signal of interest.

## Inappropriate Use of Kappa Statistic

To determine how well the WA metrics match air pollution exposures, the authors employ a Kappa statistic [22,23]. The Kappa statistic is used to assess inter-rater agreement of qualitative categorization. It is common in clinical applications to assess diagnostic agreement between physicians, qualitative rating of pain severity, and similar applications. It is not designed to be applied to continuous data, like air pollution measurements and WA metrics. Since, as mentioned earlier, a number of sources can contribute to air pollution measured at a monitor, the use of the Kappa statistic to test whether WA categorization matches air pollution categorization is an inappropriate application. 

Further, even if the use of the Kappa statistic was appropriate here, a paper entitled “*Misinterpretation and Misuse of the Kappa Statistic*” notes that “with continuous data grouped into ordinal categories, usually just for the convenience of the investigator, Kappa is so arbitrary it is virtually meaningless.” [24] Additionally, the Kappa statistic is highly sensitive to the distribution of the underlying data and can produce unreliable results, including giving low results when the underlying raters are actually concordant. Some recommend that the Kappa test be supplemented with a number of other tests. Alternative statistics, like running regressions, would be more suited to the authors’ intended application.

## Implications of Oil and Gas Health Impacts Research

The results from epidemiological, exposure assessment, and other health impacts studies have ramifications for public health. These types of studies can form the evidentiary basis for a number of strategies intended to protect the health of populations around O&G development. These include pollution controls, permitting decisions, and setback rules designed to establish, and then require, minimum distances between O&G wells and sensitive receptors and vulnerable populations, like schools, daycares, hospitals, and residences. Therefore, to protect public health, it is critical that studies on health impacts around O&G development are robust.

## Conclusions

Because of the unrepresentative air monitoring locations and inappropriate statistical methods, the Hess et. al. study does not improve our understanding of the residential exposures associated with O&G wells. For these same reasons, the Hess et al. study also does not provide information useful for decisions relevant to the health of communities nearby. Instead, the study only confirms well understood limitations of WA metrics at long distances that bear little relevance to the geographical scales in the existing epidemiology, or communities near O&G development. We recommend that future efforts and resources be directed at obtaining comprehensive, publicly available air pollutant emission rates from natural gas well sites suitable for modelling exposures to close-by residents, assessing exposure at relevant proximity to O&G wells, and validating and improving proximity WA models. Finally, we recommend that any future industry research in this area be done in collaboration with academic scientists and public health researchers.
